# Molecular Characteristics and Treatment of Endothelial Dysfunction in Patients with COPD: A Review Article

**DOI:** 10.3390/ijms20184329

**Published:** 2019-09-04

**Authors:** Botond Szucs, Csilla Szucs, Mate Petrekanits, Janos T. Varga

**Affiliations:** 1 PharmaFlight Research and Training Center, H-4030 Debrecen, Hungary; 2 Department of Anatomy, Histology and Embryology, Faculty of Medicine, University of Debrecen, Debrecen H-4032, Hungary; 3 Institute of Exercise Physiology and Sport Medicine, University of Physical Education, H-1123 Budapest, Hungary; 4 Department of Pulmonary Rehabilitation, National Koranyi Institute for Pulmonology, H-1121 Budapest, Hungary

**Keywords:** COPD, pulmonary rehabilitation, endothelial dysfunction, cardiovascular risk, pulmonary hypertension

## Abstract

Patients with chronic obstructive pulmonary disease (COPD) show systemic consequences, such as chronic systemic inflammation leading to changes in the airway, airway penetrability, and endothelial function. Endothelial dysfunction is characterized by a list of alterations of endothelium towards reduced vasodilation, proinflammatory state, detachment and apoptosis of endothelial cells, and development of atherosclerosis. COPD-induced endothelial dysfunction is associated with elevated cardiovascular risk. The increment of physical activities such as pulmonary rehabilitation (PR) training have a significant effect on COPD, thus, PR can be an integrative part of COPD treatment. In this narrative review the focus is on the function of endothelial inflammatory mediators [cytokines, chemokines, and cellular proteases] and pulmonary endothelial cells and endothelial dysfunction in COPD as well as the effects of dysfunction of the endothelium may play in COPD-related pulmonary hypertension. The relationship between smoking and endothelial dysfunction is also discussed. The connection between different pulmonary rehabilitation programs, arterial stiffness and pulse wave velocity (PWV) is presented. Endothelial dysfunction is a significant prognostic factor of COPD, which can be characterized by PWV. We discuss future considerations, like training programs, as an important part of the treatment that has a favorable impact on the endothelial function.

## 1. Introduction

Chronic obstructive pulmonary disease (COPD) is described as an incurable multisystemic inflammatory disease [1,2] defined by airflow obstruction, which is irreversible or partly reversible. COPD is found in 6%–8% of the population [3] with extra-pulmonary co-morbidities, such as respiratory muscle weakness, cardiovascular [4] and cardiac autonomic regulation diseases, as well as abnormal autonomic control of cardiopulmonary function [5,6,7].

**Figure 1:**
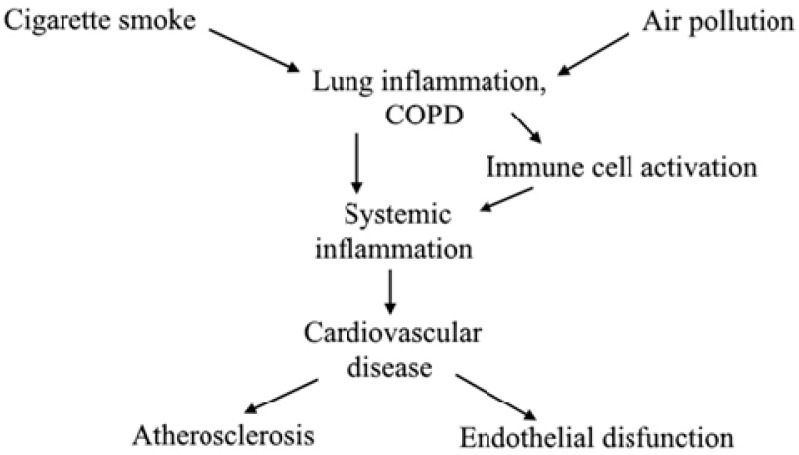
Exposure to cigarette smoke and air pollution activates immune cells [e.g., macrophages, neutrophils] which drive reactive oxygen species production and systemic inflammation. The process finally promotes cardiovascular diseases (CVD) and progression, ultimately leading to CVD-associated death.

Globally, COPD is the fourth leading cause of mortality with more than 3 million deaths per year [8]. The financial and disease burdens of healthcare utilization due to COPD in parallel with the management of its major co-morbidities, i.e., chronic cardiovascular diseases [9,10] with skeletal muscle wasting, renal function failure [11] and stroke [12,13], acute infective viral and bacterial exacerbations are also manifested [14]. COPD can be characterized with a list of pathophysiological changes triggered by exposure to inhaled pollutants, mainly to cigarette smoke. This leads to airway inflammation via the activation of pulmonary epithelial and inflammatory cells [2]. As the result of their chemotactic mediators and other inflammatory cells, like CD8+ T cells, neutrophils, monocytes and lymphocytes gather and induce chronic inflammation in the lung. Subsequently this inflammation is regarded to cause structural changes, obstructions and respiratory symptoms in the airway [15,16]. As an indirect consequence of the altered pulmonary milieu (hypoxia, vasoconstriction, and injury to the extracellular matrix) changes occur in the endothelium related to endothelial lining (Figure 1). This endothelium-lined vasculature slowly leads to cardiovascular diseases, like right ventricular dysfunction, pulmonary hypertension, coronary artery disease, and atherosclerosis [17]. Consequently, the treatment of COPD requires complex management with exercise training being a cardinal part of the rehabilitation [18,19]. It is well known that exercise training during pulmonary rehabilitation induces positive influence on the circulation, metabolism, muscles, and the overall quality of life [20,21,22,23].

In this narrative review we conducted PubMed searches to summarize and evaluate characteristics and treatment of endothelial dysfunction associated with COPD. During the database search controlled trials, observation studies, systematic reviews and meta analyses were monitored with search terms, like ’COPD’, ’pulmonary rehabilitation’, ’endothelial dysfunction’, ’cardiovascular risk’, and ’pulmonary hypertension’.

## 2. Important Characteristics and Mechanisms of the Pulmonary Endothelium

**Figure 2:**
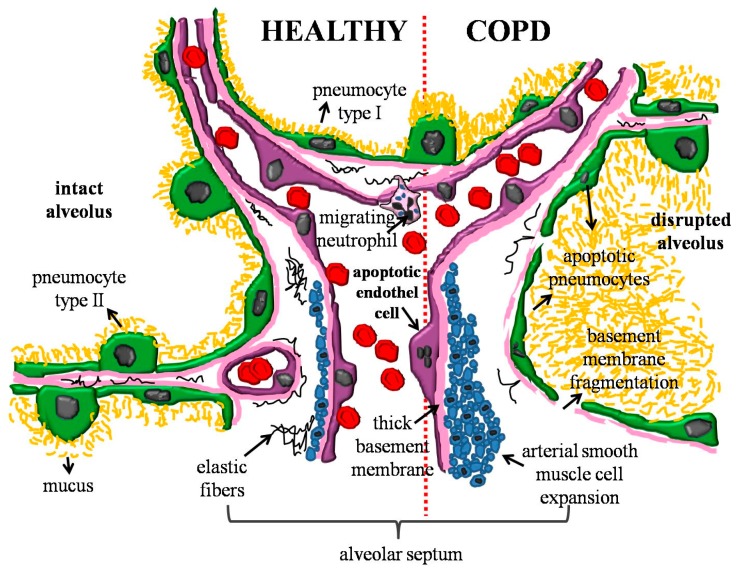
Schematic picture of healthy and COPD alveolar septum. In the COPD pulmonary tissue apoptotic pneumocytes appear in the alveolar wall and the wall becomes disrupted. The basement membrane of the pulmonary epithelium is fragmented. As the result of the enhanced enzyme activity the amount of elastic fibers is decreased. The pulmonary vasculature is thickened with arterial smooth muscle cells.

The pulmonary vasculature is lined by endothelial cells, which form a continuous single cell layer [24] (Figure 2). These endothelial cells produce a thin extracellular layer that mainly consists of collagen, proteoglycans, and laminins, and it is referred to as the basement membrane [25]. Interestingly, renal endothelial basement membrane frequently has double contour due to repetitive renal endothelial cell injury [26], less is known about the thickness of the basement membrane belonging to pulmonary vessels. On the other hand, the basement membrane of the pulmonary epithelium is more strongly characterized: in 2007 and 2009 articles described that COPD basement membrane is thicker than in healthy individuals [27,28]. Another research lab reported that the inferior portion of the basement membrane, the reticular membrane, is fragmented, variable in thickness, and more vascularized in COPD biopsies [29,30,31]. Within the basement membrane, a non-continuous layer of pericytes is embedded, whose function is to mediate endothelial cell proliferation and angiogenesis [32,33]. On the luminal part, a structure of proteoglycans and glycoproteins called glycocalyces covers the endothelial cells, and this network is involved in cell signaling and hemostasis [34]. A schematic picture of the pulmonary endothelium is presented in Figure 2. Apoptotic endothelial cells appear in the vascular wall [35] with fragmented nucleoli [36]. Consequently, the blood supply to these pulmonary septa is further limited, enhancing emphysematous morphology. The proliferation rate of smooth muscle cells is enhanced, creating a thicker vessel wall [26]. As a result of the enhanced matrix metalloproteinase activity, such as elastase, matrix metalloproteinase 1, 7, 9, 10, 12 and 28 [37,38,39] the alveolar wall loses its elasticity and finally collapses. Moreover, the alveolar epithelial cells can be characterized by elevated apoptotic rate leading to interrupted epithelial lining and disrupted alveoli [40]. Surprisingly, the increased level of proliferating cells was also reported by a research group [41,42] but the net result is the destruction of the pulmonary tissue and the development of emphysema [42]. Since the junctional complexes between endothelial cells are weakened, vascular leakage occurs between endothelial cells and the extravasation of extracellular protein may promote viscous mucus production and the formation of luminal mucus plugs [43,44]. The significance of COPD related endothelium research is shown by the fact that this airway endothelium could be a novel therapeutic target, since glucocorticosteroids can totally or partly regenerate physiological endothelium-dependent vasodilation in COPD patients [45].

## 3. Inflammatory Cells and Mediators in COPD

### 3.1. Neutrophils’ Migration and Netosis

**Figure 3:**
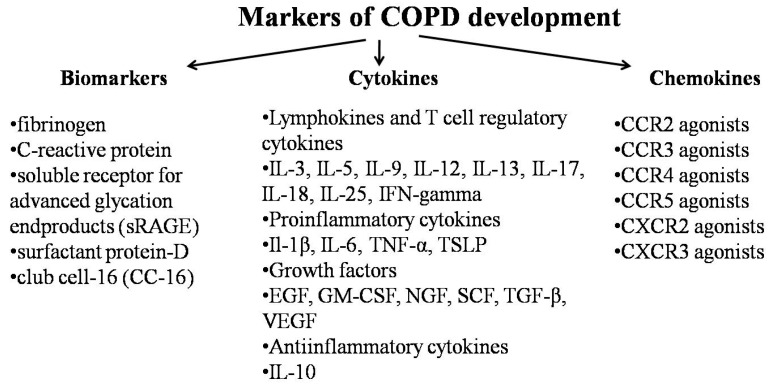
Classification of the most frequently listed markers during COPD.

Accumulating evidence suggests that inflammation is a turning point in initiating vessel remodeling in COPD [46]. COPD is related to the premature activation of epithelial and innate immune cells, such as neutrophils, eosinophils, macrophages with inflammatory mediators, comprising lipid mediators, inflammatory peptides, reactive oxygen and nitrogen species, chemokines, cytokines growth factors, and cellular proteases in patients with COPD [47,48,49]. Neutrophils are the most numerous effector cells of innate immune system with surprisingly high heterogeneity and novel cellular processes, like extracellular trap (NET) formation and microvesicle (MV) release [50,51]. The process of neutrophils generating NETs as the result of danger signal is called NETosis. Neutrophils release their protease and histone coated sticky chromatin fibers to catch and trap bacteria, fungi, and other pathogens [50]. NETs are present in a significant number of COPD patients [52,53] and just recently turned out that their amount correlates with disease severity [54,55], moreover, NET formation has been associated with ongoing airway epithelial and endothelial cytotoxicity [56]. The pathophysiology of NET in inflammatory diseases is excellently described in a review by Bonaventura et al. [57]. Regarding neutrophils, there are two ways of describing their migration towards the abluminal side [58]: first, they may connect to the apical domain of the endothelial cells via cell surface proteins, and then they migrate with the help of their cellular processes called pseudopods over one or between two endothelial cells. In case of two endothelial cells, the process is called as paracellular transmigration [59]. Nevertheless, neutrophils can also move through one endothelial cell, which is called transendothelial migration (TEM) (Figure 3). TEM has a crucial function in the inflammatory response in patients, since the previously mentioned cell surface proteins leak into the inflamed tissue after TEM and become detectable in the serum [58]. For instance, during TEM, Macrophage-1 Antigen (MAC-1) is upregulated in neutrophils during COPD [59]. Since MAC-1 string to Endothelial Intracellular Adhesion Molecule-1 (ICAM-1), this mechanism may be clinically relevant since the serum level of ICAM-1 has negative correlation to lung function [60,61]. Recently it has been published that ICAM-1 might prevent COPD exacerbations [62] The Endothelial-Leukocyte Adhesion Molecule-1 (ELAM-1) is also incorporated in TEM. ELAM-1 may be elevated in the serum of patients with COPD. These data support the role of adhesion molecules in the inflammation of the lungs and the pathogenesis of COPD [63]. So far, the general consideration about neutrophils was that they do not recirculate. After recruitment into tissue, they do their job and die within the stroma. However, recently it was postulated that a small percentage of neutrophils can migrate retrograde direction across endothelial cells [64,65]. Some of the surviving neutrophils migrate from abluminal-to-luminal direction, through endothelial cells: reverse transendothelial migration (rTEM), and they probably mediate systemic dissemination of the local inflammatory response. Meanwhile, the rest of the reversely wandering neutrophils migrate back to the interstitium and facilitate inflammation resolution [66,67].

### 3.2. Macrophages and Eosinophils

Since COPD is a heterogeneous condition with different cell types, the responsiveness to pharmacological treatments shows high variability. During COPD development, the number of lung-resident macrophages was reported to increase dramatically. These cells with neutrophils contribute to disease pathology via the production of extracellular matrix degrading enzymes [68]. During the disease, alveolar macrophages have dysregulated capacity to secrete proinflammatory mediators and proteases, induce oxidative stress, engulf microbes and apoptotic cells, and express surface and intracellular markers. They simply change their functional repertoire and further contribute to disease pathology in COPD. For instance, alveolar macrophages are normally non-polarized but in COPD there is a significant increase in macrophage polarization and co-expression of M1 and M2 macrophages [69]. In a mouse COPD model, deposition of M2 alveolar macrophages is elevated and the expression of TGF-β/Smad pathway is increased. This may indicate that M2 macrophages might contribute to COPD through changing of phenotype and TGF-β/Smad pathway [70,71].

The function of eosinophils in the pathogenesis of COPD is still covered by myth, but it seems that they can be significant biomarkers, since increased numbers of circulating eosinophils can be found in pulmonary tissue, sputum, and blood during both stable disease and exacerbations [72]. Moreover, sputum and blood eosinophil count in stable COPD patients predicts the clinical response to corticosteroids [73,74,75]. The Global initiative for the management of Obstructive Lung Disease [GOLD] recommends blood eosinophil counts as a biomarker to help guide inhaled corticosteroid used in clinical practice [74].

### 3.3. Inflammatory Mediators

Structural and inflammatory cells produce inflammatory mediators, like certain lipids, free radicals, cytokines, chemokines, and growth factors [47]. However, the amount of these mediators is elevated during the disease development, reaching the circulation and leading to systemic inflammation and via the vasculature potentiating endothelial dysfunction. There are well known lipid mediators in COPD, including PGE_2_, PGF_2α_ leukotriene B4, whose level is significantly increased compared to that of healthy individuals [76].

Other inflammatory mediators, like matrix metalloproteinases (MMP), also augment inflammation by generating chemotactic peptides, which are potent neutrophil chemoattractants. Physiological renewal, maintaining healthy tissue or the formation of pathological changes in tissue morphology demand two group of proteins: matrix metalloproteinases (MMP) and their inhibitors. Little is known about the effect of MMPs in small airway remodeling, but it seems that MMP-12 appears to play a consistent and important role in the development of emphysema [77]. This enzyme directly breaks down elastin, whereas other MMPs, particularly MMP-10 and MMP-28 promote emphysema by influencing the proteolytic and inflammatory activities of macrophages [37]. Navratilova et al. reported that MMP-9 has been repeatedly observed to be dysregulated at both the local and systemic levels in COPD patients, therefore it can be a novel prognostic biomarker [78].

Biomarkers are essential to assess the risk factors of COPD or the severity of its course. There are well known COPD correlated biomarkers like fibrinogen [79], C-reactive protein [80,81], soluble receptor for advanced glycation end products (sRAGE) [82], surfactant protein-D [83], and club cell-16 (CC16) [84] (Figure 3).

Obviously, research on chemokines and related receptors as more recently classified biomarkers could provide novel strategies of treatment since multiple studies have shown the pivotal function of chemokines in the evolvement and advancement of COPD. Chemokines make up a smaller group of cytokines which are responsible for chemotaxis of the nearby located cells, leukocyte degranulation [85], hematopoiesis [86], and angiogenesis [87]. Chemokines are best discussed in the light of the receptors to which they bind. There are some outstanding reviews describing cytokines and their association with the development of COPD [88,89,90]. Chemokine receptor expression characterizes the sensation and response of the cell to chemokine concentration gradient. Henrot and coworkers propose that the concentration gradient mainly depends on the status of the chemokines: being in fluid phase or being immobilized to ECM molecules like glycosaminoglycanes (GAGs). Moreover, they also suggest that the chemokine level also changes within the tissue and between different tissues in gross [47]. In peripheral circulation, for instance, chemokines can be manifested in solution, and they are stayed on the endothelial cell area, where they can facilitate leukocyte arrest and extravasation. Less attention has been paid to the chemokine gradient, though it’s worth keeping in mind. These chemokine ligands bind to receptors classified into major groups, such as receptors for CC chemokines CCR, receptors for CXC chemokines [CXCR], and the receptor for the lone CXC3 chemokine [89]. Based on preclinical and clinical evidence, Henrot et al. suggest that CXCL8-CXCR1/2, CXCL9/10/11-CXCR3, CCL2-CCR2, and CXCL12/CXCR4 have relationship with the pathophysiology of COPD [47]. There might be other key chemokine players of COPD like the CCL11-CCR3 axis related to eosinophil trafficking [47].

Recently a new group of potential COPD markers have appeared: the adipo(cyto)kines. Adipose tissue seems to interfere with systemic inflammation in COPD patients by producing high number of adipokines [90]. Besides being an energy storage site, the adipose tissue is an active producer of mediators involved in inflammation [91]. Moreover, adiponectine, for instance, is associated with cardiovascular outcomes in COPD patients [92]. Adiponectin possesses anti-inflammatory properties by reducing inflammatory mediators, while leptin has an opposing effect; it plays an important role in up-regulating the inflammatory system [93,94]. Oh et al. described that an increased plasma leptin and leptin/adiponectin ratio was significantly associated with change in percent emphysema over three years, proposing a potential role as a biomarker in emphysema progression in patients with COPD [94]. Conflicting findings were partially explained by a Japanese study regarding the role of adiponectin in asthma, since they monitored the isoforms of adiponectin with different molecular weight and found associations between isoforms and asthma [95]. Moreover, an article published two years ago goes further while explaining the role of adiponectin in an animal study: they come to the conclusion that adiponectin might effectively ameliorate the progression of COPD via inhibiting the endoplasmic reticulum stress-induced alveolar epithelial apoptosis [96]. Other possible markers of COPD progression are published, like CTRP-5 or ghrelin, the hunger peptide [91,97]. The latter is suggested to ameliorate respiratory function and emphysema in a dose-dependent manner [97].

Since several chemokines are identified in the manifestation of COPD [47,89] as a treatment option, neutralization of chemokines and their receptors could be applied [98,99], but there might be other approaches like interfering with chemokine-GAG interaction [100] or designing specific chemokine heterodimer agonists [101] as well. In the future, the predictive value of other important chemokines should be targeted.

## 4. Endothelial Dysfunction and Apoptosis

**Figure 4:**
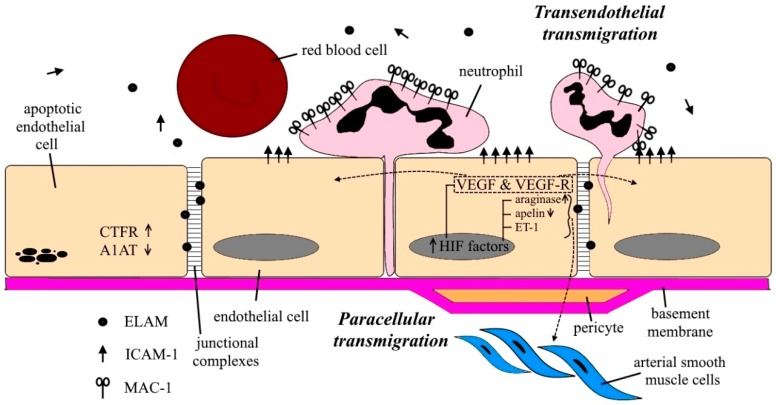
Crosstalk of the pulmonary endothelium in COPD. Neutrophils migrate between endothelial cells and this process is called paracellular migration, while when it happens via one endothelial cell, it is called transendothelial migration (TEM). Macrophage-1 Antigen (MAC-1) is upregulated in neutrophils and MAC-1 binds to Endothelial Intracellular Adhesion Molecules-1 (ICAM-1). Serum level of ICAM-1 is associated with emphysema. Endothelial-Leukocyte Adhesion Molecule-1 (ELAM-1) is also involved in TEM and upregulated in the serum of COPD patients. As the result of decreased oxygen level in the pulmonary tissue Hypoxia Induced Factors (HIF) are activated and enhance the transcription of certain target genes, such as Platelet-Derived Growth Factor β (PDGFβ) which elevates the proliferation rate of arterial smooth muscle cells. These pulmonary endothelial cells also release endothelin-1 (ET-1) and araginase1–2 which are responsible for the vasoconstriction of the arterial smooth muscle cells. The endothelial apelin inhibits vasodilation. And VEGF produced by endothelial cells acts as an autocrine and paracrine factor via VEGF receptors (VEGFR) and promotes angiogenesis and enhances intercellular junctions.

During the early stages of COPD syndrome, as a result of direct toxic injury because of the effects of smoking on the lung vessels, and then, in advanced COPD, hypoxia-mediated lung vessel remodeling might develop. At the end of the 1950s, histological reports of emphysematous lungs showed that the alveolar septa in centrilobular emphysema is considerably thin and has nearly no vasculature. First, Liebow marked that because of the drop of the circulation in the small pre-capillary blood vessels induces the dissolve of the alveolar septa [102]. Almost half a century later, Peinado described that the endothelium of the pulmonary artery in COPD patients can be characterized by morphological abnormalities [13]. Peinado performed an important experiment by measuring endothelium dependent relaxation mediated by nitric oxide in vitro in pulmonary artery (PA) rings exposed to cumulative concentrations of acetylcholine (Ach) and ADP. They found that in COPD patients, PA had lower maximal relaxation than smokers and nonsmokers and a trend towards reduced relaxation to Ach. Also, these PA had thicker intimas, especially in small arteries in smokers and COPD patients. The finding reinforces that structural alterations of the intimal layer in small pulmonary arteries may be an early feature in COPD and cigarette smoke plays a central role in the pathogenesis of structural and functional alterations of the pulmonary vasculature in COPD [13] The same research group further detected endothelial apoptosis and endothelial dysfunction in the lungs of COPD/emphysema patients [103,104,105], and the manifestation of endothelial apoptosis can be visualized in computer tomography scans, where reduced and remodeled peripheral vasculature of the lungs with COPD is revealed [106]. Structural and functional injury of the small pulmonary arteries can be seen in the early phase of COPD with vessel wall thickening, endothelial dysfunction, vascular smooth muscle proliferation, and inflammatory cell infiltration [107]. During the progression of the disease, apoptosis may occur in endothelial cells [104,108]. The common effect of these pathological processes may be related to the evolution of pulmonary hypertension and right ventricular dysfunction. Previously, researchers paid less attention to the pulmonary endothelium, but more and more data are accumulated to support the importance of the endothelium during COPD pathogenesis. One study described that apoptotic endothelial cells in COPD patients are generated by the Cystic Fibrosis Transmembrane Regulator (CTFR) protein [109]. CTFR is an anion channel expressed in both epithelial and endothelial cells, which regulates the organization of tight junctions between epithelial cells and has also been implicated in the transport of sphingosine-1 phosphate (S1P), a vascular barrier–enhancing sphingolipid [110]. CFTR function is required for stress-induced apoptosis in lung endothelial cells by maintaining adequate intracellular acidification and ceramide activation [110]. After staurosporine or hydrogen peroxide treatment, the level of endothelial apoptosis is reduced by the inhibition of CTFR [109]. Endothelial apoptosis resulting in vascular atrophy may lead to the injury of alveoli, and the evolvement of emphysema. Cigarette smoke (CS) reduces the activity of alpha-1-antitrypsin (A1AT) via posttranslational modification implying that the presence of this protein may also be important in the development of emphysema [111] (Figure 4). Taggart and his colleagues have found in vitro that A1AT prevents involvement of caspase-3 and apoptosis in pulmonary endothelial cells [111].

Extracellular vesicles also gain more and more attention in COPD associated endothelial dysfunction. Activated or apoptotic endothelial cells release endothelial microparticles into the circulation, and these extracellular vesicles are involved in paracrine and endocrine factors related to the pathway of COPD [112]. According to Lockett et al., uninjured endothelial cells transport α1-antitrypsin into the alveolar epithelial cells via endothelial cell-dependent extracellular vesicles [113]. Nonetheless, as the result of endothelial apoptosis, the endothelial transport is inhibited, which leads to the reduction of α1-antitrypsin.

As a consequence of a low level of oxygen, a certain endothelial signaling cascade is initiated. Hypoxia-Inducible Factors (HIF), for instance, are transcriptional factors that are activated in response to reduced oxygen level (Figure 3). They influence cell metabolism, survival, and angiogenesis. Both HIF-1 and HIF-2 are expressed in the pulmonary endothelium [114,115,116]. Several HIF-1 target genes are responsible for regulating angiogenesis, vascular remodeling and glucose metabolism [117]. As the result of HIF-1 activity, Platelet Derived Growth Factor β (PDGFβ) is released in the endothelium, and promotes pulmonary arterial smooth muscle expansion in general [118]. However, it should be considered that hypoxia is linked to inflammation via HIF-1 [119,120]. Endothelin 1 (ET-1), a potent vasoconstrictor, is a well recognized HIF-2 dependent gene in pulmonary tissues [121,122,123]. This transcription factor also induces vasoconstriction via other pathways, such as induction of arginase activity, since arginase 1 and 2 have been implicated in vascular remodeling by reducing the nitric oxide availability in the airways. HIF-2 also downregulates apelin; therefore, it decreases apelin-dependent vasodilation [116,124,125]. Vascular Endothelial Growth Factor (VEGF), the manifestation of which is mediated by HIF-1, is an important factor of angiogenesis and vascular permeability [126]. VEGF, just like HIF-1 expression, is up-regulated by hypoxia [127]. It has been proved to be related to the pathway of COPD, and HIF-1α-regulated VEGF overexpression may be an important factor of chronic bronchitis [128]. VEGF and its receptors VEGFR-1 (Flt-1) and VEGFR-2 (KDR/Flk-1) could be involved in tissue remodeling and the angiogenesis of COPD [86]. Santos et al. have observed that VEGF expression varies according to the severity of the disease. In the early phase of COPD, vascular remodeling induced by poorly differentiated smooth muscle cells with high level of VEGF, however in a severe stage, the remodeling is confirmed by differentiated smooth muscle cells producing fewer VEGF [129]. Certain VEGF isoforms seem to be responsible for vessel development and maintenance [129], and the reduction in the number of certain VEGF isoforms might be responsible for the apoptosis that plays a role in the alveolar septa of the emphysematous lungs [104,129]. To replace the injured endothelial cells, increased proliferation characterizes the endothelium, which finally leads to the depletion of endothelial cells. These senescent cells are unable to progress through the cell cycle; however, they remain metabolically functional [130]. Telomer shortening also characterizes these cells [131]. Moreover, inflammatory markers are released by senescent pulmonary endothelial cells and may trigger further inflammation in COPD patients [132].

## 5. Dysfunction of the Endothelium and its Effects in COPD Patients: Pulmonary Hypertension

Endothelial dysfunction is described as injured endothelial dependent vasodilatation resulting from the breakdown of intravascular endothelial septum and loss of the anti-adhesive and antithrombotic functions of the endothelium [133]. It is well known that endothelial dysfunction can be manifested in the pulmonary arteries of end stage patients with COPD, and this might be related to the evolvement of pulmonary arterial hypertension [PAH] [105]. PAH has traditionally been defined by a mean pulmonary artery pressure of greater than 25 mmHg, there are five different groups of PAH based on different causes [134]. In this review, we are focusing on Group3: PAH due to lung disease [135]. Mild to moderate PAH can be related to higher mortality and is present in 50% of the patients [136]. PAH is also known to be associated with a weaker exercise capacity [137] and a frequent exacerbator phenotype [138]. About half of the COPD patients develop PAH during physical exertion due to hypoxic pulmonary arterial vasoconstriction [139]. Pulmonary hypertension in COPD is driven by hypoxic vasoconstriction, systemic inflammation, endothelial dysfunction, and polycythemia, as well as persistent lung inflammation and impaired lung function. The factors that drive pulmonary hypertension are the following: systemic inflammation, impaired lung function accompanied by lung inflammation, and hypoxic vasoconstriction. All of these factors promote remodeling of the pulmonary arterioles, causing them to narrow and resulting in subsequent increases in pulmonary blood pressure (BP) and worsening in hypoxia [140]. Increased pulmonary pressure is known to cause structural change of the right part of the heart, manifested in *cor pulmonale*, which often leads to ventricular failure due to the excess strain put on the heart muscle. This persistent pulmonary inflammatory response is driven by the increment of reactive oxygen species (ROS) and reactive nitrogen species (RNS) in COPD patients; thus, pulmonary artery stiffness, pulmonary hypertension, and *cor pulmonale* are often associated with respiratory diseases, such as COPD [141,142]. A schematic presentation of lung inflammation and the pathogenesis of COPD and comorbidities can be seen in Figure 1.

In idiopathic PAH (iPAH), the deposition of extracellular matrix proteins can be seen in the pulmonary arteries such as in patients with COPD and an intimal manifestation of poorly differentiated smooth muscle cells. Endothelial dysfunction can be manifested in the early phase of both group 1 and group 3 pulmonary hypertension (secondary to COPD), and it seems to contribute to the advancement of both diseases [143].

## 6. Smoking and Endothelial Dysfunction

Smokers and early stage COPD patients show evidence of endothelial dysfunction [144]. In COPD, the endothelium dependent relaxation is reduced, and small arteries show thickening both in smokers and in patients with COPD [47]. Clinical data proved the development of emphysema and PAH by endothelial dysfunction [145]. Induced endothelial dysfunction and injuries can be developed by acute cigarette smoke (CS) exposition: CS exacerbates acute lung injury in vivo in mice, and CS increases the permeability of endothelial monolayers in vitro [146]. CS also enhances granulocyte production, causing neutrophilic infiltration into the lungs in response to neutrophil chemotactic factors [147]. An increase in eosinophilic infiltration and inflammation drives the recruitment of neutrophils and other associated inflammatory cells into the lung, thus exacerbating the persistent pulmonary inflammation [61]. The number of activated neutrophils is increased in the bronchoalveolar lavage fluid (BALF) and the sputum of COPD patients, and it has a direct relationship with the degree of the disease. These neutrophils are activated by sputum supernatant and granule proteins that are up-regulated in the lungs of patients with COPD [147,148]. Activated neutrophils secrete proteases that drive alveolar destruction. During acute exacerbations of COPD, neutrophilic inflammation is further enhanced, promoting excessive degradation of the lung parenchyma [47].CS exposure increases the permeability of the alveolar capillary layer and components of CS (nicotine and its metabolites, acrolein, superoxide anion or hydroxyl radicals) to get into the circulation, where they can interact with and injured endothelial cells of the pulmonary and systemic circulations [26]. These findings clearly indicate that CS exposure damages endothelial cells in vitro; nevertheless, these findings are ignored or treated clinically irrelevant apart from the fact that they support the idea that emphysema is, to some extent, a vascular abnormality. The “vascular COPD” phenotype is an under-recognized clinical entity; endothelial dysfunction and injury could be a pathological background related to COPD co-morbidities with the development of emphysema [149].

It must be noted that the pulmonary vascular remodeling resulting in pulmonary hypertension can develop independently from the parenchymal destruction and the loss of lung vessels. These changes result in the formation of neointimal lesions completely different from vascular alterations in highlanders and the hypoxia induced models. The presence and absence of these lesions attribute to the distinction between pathological features of PAH and COPD and are composed of phenotypically altered and proliferating endothelial cells [150].

Although there is little clinical research on new-generation heat-not-burn cigarettes in comparison with electronic vaping cigarettes and traditional tobacco combustion cigarettes, a recent study showed that acute effects of these are severely different on platelet function, cardiovascular attributes and antioxidant reserve, with traditional tobacco combusting cigarettes showing the most disadvantageous effects in randomized cross-over trials [151].

## 7. Arterial Stiffness, Aortic Pulse wave Velocity and Pulmonary Rehabilitation

Arterial stiffness characterized by aortic pulse wave velocity (PWV) can be used as a prognostic factor in risk stratification of cardiovascular diseases and mortality in some selected groups of patients [152]. A higher rate of aortic stiffness can be determined in COPD [152], even with no co-morbidities like diabetes mellitus or a cardiovascular disease. An increment of arterial stiffness has a lot of non-atherosclerotic outcome, such as renal, cardiac, and other vascular injuries [153,154].

Structural and functional factors as well as the distending pressure and mean arterial blood pressure (MAP) have an influence on aortic PVW. Clinical data supports of the effect of chronic inflammation [155] for the functional and structural factors, and interventional anti-inflammatory trials determined the favorable effect on aortic stiffness in other inflammatory processes. In COPD patients, strong clinical data supported the relationship between aortic stiffness and systemic inflammatory factors [156]. The administration of anti-inflammatories may be an option to improve aortic stiffness in COPD.

We observed pathological arterial stiffness as a common observation in our patients regarding the ability of the patients that were in the elevated/pathological threshold group at the onset of the rehabilitation program [157]. The rehabilitation was based on respiratory training in the morning, chest wall mobilization, learning controlled breathing techniques (30 min), inhalation, expectoration, and smoking cessation. The training was individualized as continuous or interval types of cycle- and/or treadmill 2–3 times daily, lasting for 20–30 min in a four-week period. One of the main focuses was to determine the vascular function and the effectiveness of pulmonary rehabilitation in terms of endothelial function [157]. The augmentation index (AIX) in more than half of the patients improved. A clear improvement tendency was also observed in blood pressure. Abnormal elasticity was observed by high PVW, and there was significant improvement as a result of physical activity. Rehabilitation was also effective according to the functional markers. The results of the four-week program are visible in Table 1.

There is limited information about COPD and the significant reduction in aortic PWV in terms of exercise, including pulmonary rehabilitation [23,158,159].

Vanfleteen and coworkers have determined a significant variability in the change in aortic PWV as result of pulmonary rehabilitation [160]. Some patients had a significant reduction of aortic PWV after pulmonary rehabilitation, others did not [160]. There was clinical importance of understanding the potential mechanisms, the pharmacological and non-pharmacological causes of higher levels of aortic PWV in COPD patients [160]. Randomized, controlled trials with a large population are necessary to resolve the question about effectiveness in the future.

## 8. Conclusion and Future Perspectives

Endothelium of COPD patients is characterized by functional and morphological abnormalities. The reduced and remodeled peripheral vasculature of the lung is related to pulmonary hypertension and right ventricular dysfunction. The factors that drive pulmonary hypertension are the following: systemic inflammation, impaired lung function accompanied by lung inflammation, and hypoxic vasoconstriction.

Endothelial dysfunction in COPD is driven by several factors: premature activation of immune cells, inflammatory mediators and peptides, reactive oxygen and nitrogen species, chemokines, cytokines, etc. The migration of the neutrophils has a crucial and clinically relevant role in the inflammatory response and the pathogenesis of COPD in patients. Biomarkers like fibrinogen, C-reactive protein, sRAGE, surfactant protein-D, and club cell protein-16 are essential for assessing the risk factors of COPD, while research could provide novel strategies for treatment, like neutralization of chemokines with chemokine-GAG interactions or special heterodimer agonists for chemokines. In the future, predictive values of chemokines should also be targeted.

Smokers and early stage COPD patients show evidence of endothelial dysfunction, and thar cigarette smoke exposure damages endothelial cells in vitro; nevertheless, these findings are ignored or treated as being clinically irrelevant apart from the fact that they support the idea that emphysema is, to some extent, a vascular abnormality. Future research should look deeper into the vascular COPD phenotype, since it is an under recognized clinical entity. Endothelial dysfunction and injury could have a pathological background with the comorbidities of COPD in the development of emphysema. Little is known about the impact of vaping and heat-not-burn cigarettes; this could be a very interesting research topic, as these devices are becoming more and more popular.

As highlighted before, the significance of COPD related endothelium research is great. This airway endothelium could be a novel therapeutic target, since glucocorticosteroids can partly or regenerate endothelium-dependent vasodilation in COPD patients.

Recent studies show that pulmonary rehabilitation focusing on physical activity has a very advantageous effect on endothelial function and arterial stiffness. Still rehabilitation has to be a focus of future research because it can be a very effective tool in alleviating discomfort during the treatment of COPD.

## Figures and Tables

**Table 1:** Characteristics measured by Arteriograph and functional and quality of life marker parameters during a 4 week rehabilitation period (Sys: systolic blood pressure; Dias: diastolic blood pressure; AIX: augmentation index; PWV: pulse wave velocity; DAI: diastolic area index, FEV1: forced expiratory volume in the first second; FVC: forced vital capacity; mMRC: modified Medical Research Council Dyspnea Scale; MIP: maximal inspiratory pressure; CWE: chest wall expansion; BHT: breath holding time; GS: grip strength; 6MWD: 6 min walking distance; CAT: COPD assessment test; *p* < 0.05).

Parameter	Before Treatment	After Treatment
Sys (Hgmm)	133.38 ± 22.15	126.48 ± 20.22
Dias (Hgmm)	76.95 ± 14.37	75.4 ±12.7
Pulse (bpm)	76.95 ± 14.37	72.53 ± 13.65
AIX (%)	3.54 ± 35.59	2.93 ± 30.79
PWV (m/s)	11.74 ± 2.13	11.4 ± 2.73
DAI (%)	46.32 ± 6.81	47.1 ± 70.2
FEV1 (l)	45.43 ± 20.2	45.06 ± 18.2
FVC (l)	75.81 ± 22.71	74.78 ± 17.37
mMRC	1.86 ± 0.71	1.63 ± 0.6 *
MIP (cmH2O)	57.72 ± 22.69	63.63 ± 18.01 *
CWE (cm)	2.84 ± 1.26	4.00 ± 1.76 *
BHT (s)	25.77 ± 10.63	29.21 ± 11.60 *
GS (kg)	24.87 ± 11.88	27.03 ± 11.43 *
6MWD (m)	335.32 ± 110.43	398.32 ± 126.21 *
CAT	17.00 ± 8.49	11.89 ± 7.31 *
